# Molecular Epidemiology and Antimicrobial Resistance of *Haemophilus influenzae* in Adult Patients in Shanghai, China

**DOI:** 10.3389/fpubh.2020.00095

**Published:** 2020-03-27

**Authors:** Xin-Xin Li, Shu-Zhen Xiao, Fei-Fei Gu, Wei-Ping He, Yu-Xing Ni, Li-Zhong Han

**Affiliations:** Department of Clinical Microbiology, Ruijin Hospital, Shanghai Jiao Tong University School of Medicine, Shanghai, China

**Keywords:** *Haemophilus influenzae*, molecular epidemiology, drug resistance, multilocus sequence type, FtsI gene

## Abstract

**Background:** The serotype and antimicrobial resistance of *Haemophilus influenzae* in adult patients have changed due to the application of antimicrobials and *H. influenzae* type b (Hib) vaccine worldwide. However, the epidemiologic characteristics of *H. influenzae* in Shanghai are still unavailable.

**Objective:** To determine the serotype distribution, antimicrobial resistance and multilocus sequence type (MLST) of *H. influenzae* in adult patients in Shanghai.

**Methods:** A total of 51 clinical isolates from adult patients were consecutively collected. Serotypes were determined according to specific capsule gene, *bexA*, amplified by PCR. Antimicrobial susceptibility test was carried out by the broth microdilution method. β-lactamase production was detected by cefinase disk and the *ftsI* gene were amplified and sequenced to determine the penicillin binding protein 3 (PBP3) mutation. Molecular epidemiology was performed by MLST analyses.

**Results:** All isolates studied were nontypeable *H. influenzae* (NTHi) and three of them (5.88%) caused invasive infection. The resistant rates of ampicillin and trimethoprim/sulfamethoxazole were both 45.10%. One third of these isolates produced TEM-1 type β-lactamase and 11.76% were β-lactamase negative ampicillin resistant strains (BLNAR). The PBP3 mutation was detected in 74.51% of the isolates, of which 12 belonged to group III. A total of 36 sequence types (STs) were identified among all isolates. Four isolates of ST103 (7.84%) all produced β-lactamase without mutation of PBP3.

**Conclusion:** *H. influenzae* infections among adults in Shanghai are predominately caused by NTHi with genetic diversity among adult patients. The prevalence of both β-lactamase production and PBP3 mutation may contribute to high ampicillin resistance rate in Shanghai.

## Introduction

*Haemophilus influenzae* is a commensal bacteria colonizing mostly the upper respiratory tract causing mucosal and severe invasive infections such as meningitis and septicemia (1). Hence, *H. influenzae* is considered a priority pathogen by WHO (2). It is characterized as serotype a to f based on polysaccharide capsule antigens and lack of a capsule are identified as nontypeable (NTHi). NTHi has been the most prevalent cause of community-acquired pneumonia and exacerbation of chronic obstructive pulmonary disease (COPD) (3, 4). In addition, invasive diseases caused by NTHi are emerging globally and is predisposed by age and coexisting conditions (5). It is more likely that NTHi strains, like many other opportunists, are increasing due to increased numbers of individual with coexisting conditions and intensified diagnostics among them.

β-lactam antibiotics are the first choice of *H. influenzae* treatment. Ampicillin resistance of *H. influenzae* can utilize two mechanisms of action. β-lactamase production is the dominant mechanism, which is encoded most often by *bla*_TEM−1_ and rare *bla*_ROB−1_ (6). Strains that are resistant to ampicillin due to producing β-lactamase are termed β-lactamase positive, ampicillin resistant (BLPAR). The second mechanism is amino acid substitution of the transpeptidase enzyme, PBP3, encoded by the *ftsI* gene (7). Strains which do not produce β-lactamase but are resistant or intermediate to ampicillin are termed β-lactamase negative, ampicillin resistant (BLNAR) or β-lactamase negative, ampicillin intermediate (BLNAI) according to the breakpoints for ampicillin (8). In addition, strains possessing both mechanisms are termed β-lactamase positive, amoxillin-clavulanate resistant (BLPACR) (9). However, strains with mutation of PBP3 may not show resistance to ampicillin or amoxillin-clavulanate, which are classified as genetically BLNAR or BLPACR (gBLNAR/gBLPACR) (8). According to Clinical and Laboratory Standards Institution (CLSI) guideline (10), BLNAR strains should be considered resistant to amoxicillin-clavulanate, ampicillin-sulbactam, cefaclor, cefamandole, cefetamet, cefonicid, cefprozil, cefuroxime, loracarbef, and piperacillin-tazobactam, despite apparent *in vitro* susceptibility of some BLNAR strains to these agents. Therefore, the classification based on resistant phenotype is essential to clinical treatment. It also useful to understand the spread of drug-resistant genes.

Surveillance of serotype distribution and prevalence of drug-resistant strains in the general population is critical for public health department of government to develop appropriate prevention protocol for *H. influenzae* infection. In this study, we preliminarily investigated the serotype distribution, antimicrobial resistance, and molecular epidemiology of *H. influenzae* among adult patients in Shanghai, China.

## Materials and Methods

### Patients and Bacterial Isolates

The investigation was conducted at Shanghai Ruijin Hospital, a university-affiliated tertiary institution with ~1,800 licensed beds, serving approximately two million outpatients per year. A total of 51 isolates (only the first strain isolated from each patient was enrolled) were consecutively collected from the department of clinical microbiology, one central laboratory of this hospital, from July, 2015 to June, 2018. All isolates were identified using matrix-assisted laser desorption ionization-time of flight mass spectrometer (bioMérieux, Marcy-l'Étoile, France) and further confirmed by detection of *fucK, omp2* gene as previously described (11). Isolates were restocked in broth supplemented with 30% (v/v) glycerol at −80°C. All isolates were incubated 18–24 h on selective chocolate plates at 35°C with 5% CO_2_.

This study was approved by the Ethics Committee at Ruijin Hospital affiliated with the School of Medicine at Shanghai Jiao Tong University. The Review Board exempted requirement for informed consent since this retrospective study used only the bacterial samples and did not have any negative impact on the patients.

### DNA Preparation

Rapid DNA extraction was performed by boiling 35 well-isolated colonies in 200 μL of sterile DNAse-free distilled water for 10 min. The suspension was centrifuged at 12,000 rpm/min for 10 min. The supernatant was transferred into a second sterile tube and stocked at −20°C until required.

### Serotyping

Serotyping was performed by amplifying capsule gene, *bexA*, using PCR as described previously (12). Strains which could not be identified by PCR were classified as nontypeable (NT).

### Antimicrobial Susceptibility Test

The minimum inhibitory concentrations (MICs) were determined by microdilution method according to the CLSI guideline (13). Eighteen antibiotics were tested (μg/mL): ampicillin (0.06–4), amoxillin-clavulanate (2/1–8/4), cefuroxime (2–16), cefepime (1–4), aztreonam (2–4), meropenem (0.12–16), azithromycin (0.25–4), tetracycline (2–8), ciprofloxacin (0.03–4), trimethoprim-sulfamethoxazole (0.12/2.4–4/76), chloramphenicol (1–16), rifampin (0.5–4), piperacillin-tazpbactam (0.5/4–2/4), lomefloxacin (1, 2), cefixime (0.5–2), ceftriaxone (0.12–2), ampicillin-sulbactam (1/0.5–4/2), ceftazidime (1–4). The results were interpreted according to CLSI standards (10). *H. influenzae* ATCC 49247 and ATCC 49766 were used for quality control.

### β-Lactamase and PBP3 Mutation Detection

Production of β-lactamase was detected by BD BBL™ Cefinase™ (BD laboratories, Franklin Lakes, NJ, USA). The *bla*_*TEM*−1_ and *bla*_*ROB*−1_ genes were identified by PCR as previously reported, respectively (14). The gene *ftsI* was sequenced and compared with that of Rd KW20 strain to determined the mutation of PBP3 (15). Isolates with PBP3 mutation patterns were classified to group I, II, III, and III-like as previously reported (9).

### Multilocus Sequence Type (MLST)

Seven housekeeping genes (*adk, atpG, frdB, fucK, mdh, pgi*, and *recA*) were amplified, sequenced, and analyzed. Alleles and sequence types (STs) were determined according to the database (https://pubmlst.org/hinfluenzae/). Sequence and STs that could not be found in the database were submitted to the curator. Clustering of related STs was analyzed by eBURST Version 3.0.

## Results

### Clinical Data

A total of 51 *H. influenzae* isolates were collected among adult patients in Ruijin Hospital, three isolates (5.88%) from blood samples were regarded as invasive infection and 48 (94.12%) from sputum samples were regarded as non-invasion. The median age of the patients in this study was 62 years old (19–83 years old) and 41.18% patients were ≥ 65 years old. The gender distribution was 34 males and 17 females. According to the initial diagnosis, malignant tumor, and cardio-cerebrovascular disease were the most common followed by pulmonary and autoimmune disease in adult patients with *H. influenzae* infection. All patients hospitalized in 20 clinical departments: 23.53% (*n* = 12) of patients were from the department of pulmonary medicine and 13.73% (*n* = 7) were from dermatology department. Others were distributed in the department of nephrology, cardiothoracic surgery departments etc. (Supplementary Table 1).

### Serotype and Antimicrobial Susceptibility

**Figure 1:**
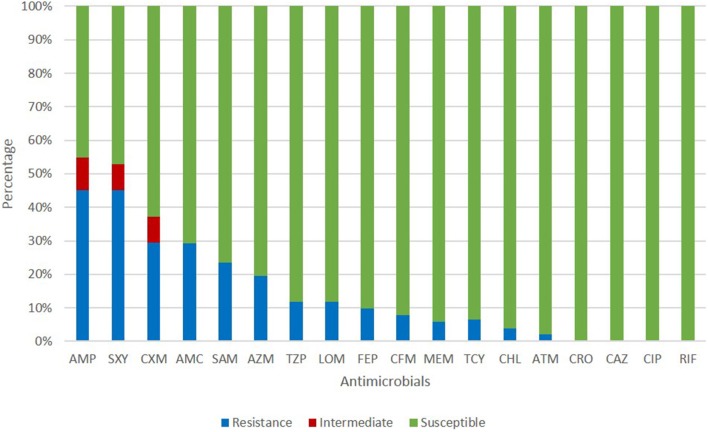
Antimicrobial susceptibility of *H. influenzae* among adults in Shanghai. AMP, ampicillin; SXT, trimethoprim-sulfamethoxazole; CXM, cefuroxime; AMC, amoxillin-clavulanate; SAM, ampicillin-sulbactam; AZM, azithromycin; TZP, piperacillin-tazpbactam; LOM, lomefloxacin; FEP, cefepime; CFM, cefixime; MEM, meropenem; TCY, tetracycline; CHL, chloramphenicol; ATM, aztreonam; CRO, ceftriaxone; CAZ, ceftazidime; CIP, ciprofloxacin; RIF, rifampin.

All isolates in this study were NTHi. The antimicrobial susceptibility of these isolates is shown in Figure 1. The resistance rates of ampicillin, trimethoprim-sulfamethoxazole, and cefuroxime were 45.10, 45.10, and 29.41%, respectively. Cefepime, cefixime, meropenem, tetracycline, chloramphenicol, and aztreonam could inhibit more than 90% of isolates. All isolates were susceptible to ceftriaxone, ceftazidime, and ciprofloxacin. The resistance pattern of *H. influenzae* isolates in the current study are shown in Table 1. Among 51 isolates, 21.57% were only resistance to β-lactam antibiotics and 19.61% were resistant to β-lactam antibiotics together with trimethoprim-sulfamethoxazole (11.76%) or azithromycin (7.84%). Seven isolates (13.73%) were resistant to three or more types of antimicrobials. The most frequent pattern of resistance was β-lactam-AZM-SXT (*n* = 4), seen in ST1218, ST1494, ST422, and ST57. The other three MDR patterns were β-lactam-TCY-CHL-LOM (ST321), β-lactam-AZM-LOM (ST503), and β-lactam-TCY-CHL-SXT (ST2105) (Table 1).

**Table 1:** Antimicrobial resistance pattern of *H. influenzae* among adults in Shanghai.

**Antibiogram**	**ST**	**N**
–	ST215, ST1069, ST107,ST1520, ST1565, ST2095, ST2099, ST2107, ST2108, ST41,	11
AMC-CXM	ST253	2
AMC-SXT	ST57	1
AMP	ST103, ST2109	4
AMP-AMC-CXM-AZM-SAM	ST422	1
AMP-AMC-CXM-FEP-MEM-AZM-SAM	ST834	1
AMP-AMC-CXM-FEP-SXT-TZP-CFM	ST107	1
AMP-AMC-CXM-FEP-TZP-SAM	ST834	1
AMP-AMC-CXM-MEM-TZP-SAM	ST12, ST2098	2
AMP-AMC-CXM-SXT-TZP-SAM	ST644	1
AMP-AMC-CXM-TCY-CHL-LOM-SAM	ST321	1
AMP-AMC-CXM-TZP-SAM	ST834	1
AMP-AZM	ST527	1
AMP-AZM-LOM	ST503	1
AMP-AZM-SAM	ST34	1
AMP-AZM-SXT	ST1218	1
AMP-AZM-SXT-SAM	ST57	1
AMP-CFM	ST103	1
AMP-CXM-FEP-ATM-AZM-SXT-CFM-SAM	ST1494	1
AMP-CXM-FEP-AZM-SXT-CFM-SAM	ST422	1
AMP-SXT	ST57	1
AMP-TCY-SXT-CHL	ST2105	1
AZM	ST586	1
CXM-SXT	ST84, ST1032	2
SXT	ST12, ST1218, ST2106, ST245, ST267, ST321, ST481	7
SXT-LOM	ST3, ST396, ST481, ST487	4

*AMP, ampicillin; SXT, trimethoprim-sulfamethoxazole; CXM, cefuroxime; AMC, amoxillin-clavulanate; SAM, ampicillin-sulbactam; AZM, azithromycin; TZP, piperacillin-tazpbactam; LOM, lomefloxacin; FEP, cefepime; CFM, cefixime; MEM, meropenem; TCY, tetracycline; CHL, chloramphenicol; ATM, aztreonam*.

### Production of β-Lactamase and PBP3 Mutation

TEM-1 type β-lactamase was produced by 17 (33.33%) of isolates and six isolates were identified as BLNAR (Table 2). Amino acid substitutions in PBP3 occurred in 38 (74.51%) isolates. The isolates accounted for the following proportions thereof: Group I 5.88% (*n* = 3), group IIa 13.72% (*n* = 7), group IIb 21.57% (*n* = 11), group IIc 5.88% (*n* = 3), group IId 3.92%, group III 23.53% (*n* = 12) and group III-like 3.92% (*n* = 2) (Table 3). Based on β-lactamase production and mutation of PBP3, 13.73% of total isolates were identified BLPAR and 5.88% were BLPACR and 43.14 and 13.73% of isolates classified as gBLNAR and gBLPACR, respectively. Cefuroxime MICs of 51 isolates ranged from ≤ 2 to >16 μg/mL. The cefuroxime MIC values in BLNAR isolates were higher than those in BLPAR isolates. Five BLNAR and three BLPACR isolates with PBP3 mutation belonged to group III or III+IIb, whilst most gBLNAR and gBLPACR isolates belonged to group I or II (Table 4).

**Table 2:** Production of β-lactamase and the susceptibility of ampicillin of *H. influenzae* among adults.

**β-lactamase**	**AMP-R**	**AMP-I**	**AMP-S**	**TEM-1^**a**^**	**ROB-1^**a**^**
+	17	0	0	17	0
–	6	5	23	–	–

a *The type of β-lactamase gene*.

**Table 3:** Amino acid substitutions identified in the *ftsI* gene of *H. influenzae* isolates.

**Group^**a**^**	**β-lactamase**	***n***	**Asp-350**	**Ser-357**	**Ala-368**	**Met-377**	**Ser-385**	**Leu-389**	**Ala-437**	**Ile-449**	**Gly-490**	**Ala-502**	**Arg-517**	**Asn-526**
I	+	1										Val	His	
	–	1										Val	His	
	+	1											His	
IIa	+	1	Asn								Glu			Lys
	–	1	Asn								Glu			Lys
	–	1									Glu			Lys
	–	3												Lys
	+	1												Lys
IIb	–	1	Asn						Ser			Val		Lys
	–	1	Asn						Ser		Glu	Val		Lys
	–	1	Asn								Glu	Val		Lys
	–	4	Asn			Ile						Val		Lys
	–	1	Asn	Asn								Val		Lys
IIc	–	1	Asn									Thr		Lys
IId	–	2								Val				Lys
III	+	1	Asn			Ile	Thr	Phe						His
	+	1	Asn	Asn		Ile	Thr	Phe						Lys
	–	5	Asn	Asn		Ile	Thr	Phe						Lys
III+IIb	+	2	Asn	Asn		Ile	Thr	Phe				Val		Lys
	–	1	Asn	Asn		Ile	Thr	Phe				Val		Lys
III+IIc	–	2	Asn	Asn		Ile	Thr	Phe				Thr		Lys
III-like	–	1	Asn	Asn		Ile	Thr						His	
	+	1	Asn	Asn		Ile	Thr	Phe			Glu		His	
Miscellaneous	–	1			Thr									
	–	1							Ser					
	+	1	Asn											
No changes		13												
		51												

a *Isolates are classified as seven groups: I,IIa, IIb, IIc, IId, III, and III-like according to previous criteria (9)*.

**Table 4:** MIC ranges of three β-lactam antibiotics in different groups of *H. influenzae*.

**Group**	**PBP3 mutation**	**n**	**MIC_**AMP**_**	**MIC_**AMC**_**	**MIC_**CXM**_**
BLNAS	No changes	6	0.12–0.5	≤2/1	≤2
BLNAR	IIb	1	4–>4	≤2/1–>8/4	16–>16
	III	4			
	III+IIb	1			
BLPAR	No changes	7	>4	≤2/1	≤2
BLPACR	III	1	>4	>8/4	>16
	III+IIb	2			
gBLNAR	I	1	0.25–2	≤2/1–>8/4	≤2–>16
	IIa	5			
	IIb	7			
	IIc	1			
	IId	2			
	III	1			
	III+IIc	2			
	III-like	1			
	Miscellaneous	2			
gBLPACR	I	2	>4	≤2–4	≤2–>16
	IIa	2			
	III	1			
	III-like	1			
	Miscellaneous	1			

*BLPAR: isolates which were β-lactamase positive, ampicillin resistant. BLNAR: isolates which were β-lactamase negative, ampicillin resistant. BLPACR: isolates which were β-lactamase positive, and PBP3 mutation together with amoxillin- clavulanate resistant. gBLNAR/gBLPACR: isolates with PBP3 mutation may not show resistant to ampicillin or amoxillin- clavulanate*.

### MLST Analysis of *H. influenzae*

**Figure 2:**
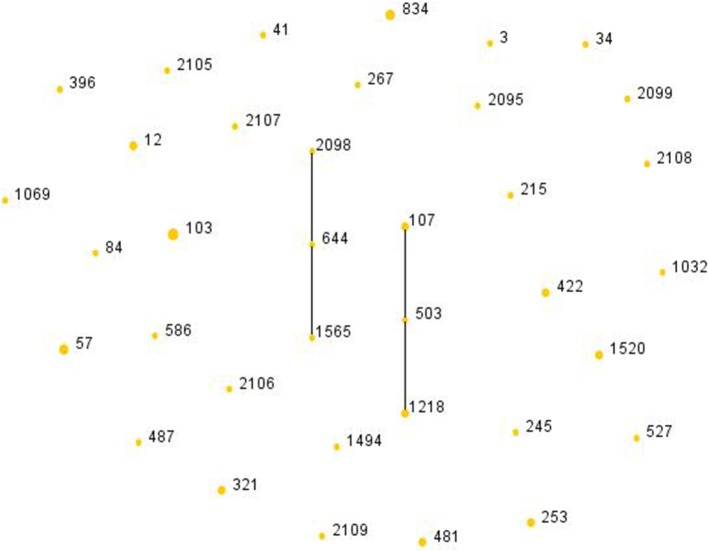
Population snapshot of *H. influenzae*. The STs were displayed as a single eBURST diagram by setting the group definition of zero of seven shared alleles. Each number represents one ST and the area of each circle indicates the prevalence of the ST in the MLST data of this study. All 36 STs were shown here in only one group. MLST, Multilocus sequence typing; ST, Sequence type.

Among the 51 isolates, 36 STs were identified as shown in Figure 2. ST103 was the most frequent type (7.84%), followed by ST57 (5.88%), ST834 (5.88%). In addition, ST2095, ST2098, ST2099, ST2105, ST2106, ST2107, ST2108, ST2109 were reported for the first time in the current study. Four ST103 isolates were all BLPAR with the same sequence of *ftsI* gene (Table 5). There were two clonal complexes (CC), one of which the primary founders were ST503 with two single-locus variants (SLVs), ST107 and ST1218. The primary founder of the other clonal complex was ST644 with two SLVs, ST1565, and ST2098.

**Table 5:** The common characteristic of ST103.

**Isolates**	**ST**	**TEM-1**	***ftsI***	**Antibiogram**
RJHH019	103	+	27	AMP-CFM
RJHH071	103	+	27	AMP
RJHH132	103	+	27	AMP
RJHH158	103	+	27	AMP

## Discussion

*H. influenzae* is a common pathogen causing infection in children under 5 years of age and adults over 65 years of age. The Hib vaccine is a cost-effective intervention among children in mainland China to protect against pneumonia, meningitis and other vaccine-preventable diseases (16). However, the Hib carriage rate in Shanghai was 8.0% in children of 12–18 months (17). Since the introduction of Hib vaccine, NTHi strains predominantly cause upper respiratory tract and severe invasive disease, and became a global concern (5, 18). Consistanly, all 51 isolates among adults enrolled in this study were serotyped as nontypeable, indicating that NTHi also predominated *H. influenzae* infection in Shanghai, which traditional Hib vaccine was unable to prevent the current *H. influenzae* infection. MLST analysis is a common method used to investigate clonal spread of a certain pathogen. There were 36 STs identified in 51 clinical isolates, with ST103 (7.84%) most commonly found. The genetic diversity of *H. influenzae* isolates showed no predominant clonal spread in Shanghai, a similar phenomenon was also demonstrated among children in Japan and England (19, 20). ST103 isolates were all non-PBP3 mutants but produced β-lactamase, similar to the results obtained in Italy (21).

In mainland China, limited surveys focused on the epidemiologic data of *H. influenzae* from children, especially under 5 years. In Beijing, the overall annual carriage rates of *H. influenzae* in children younger than 5 years of age decreased from 35.5 to 18.7% in 10 years (2000, 2002–2010, 2012). However, the production of β-lactamase increased in the 10 year period with no β-lactamase-negative ampicillin resistant strains (22). Qin et al. showed that most *H. influenzae* isolates (30/37, 81.1%) causing community-acquired pneumonia (CAP) in adults were NTHi in Shanghai in 2009. In addition, the rates of strains with β-lactamase and of BLNAR accounted for 27.0 and 29.8%, respectively (23). In our study, we found all isolates from adult patients were nontypeable, the 33.3% of the isolates enrolled produced β-lactamase and 11.76% possessing mutation in PBP3 were resistant to ampicillin, which indicated that the production of β-lactamase was increased and BLNAR was more frequent in adult patients than pediatric patients.

Amoxillin, ampicillin-sulbactam, and amoxillin-clavulanate, are usually used as the first choice for clinical therapy of *H. influenzae* infections (24). However, ampicillin-resistance has increased globally. NTHi burden and the ampicillin-resistance mediated by β-lactamase and/or PBP3 mutation increased in Italy from 2012 to 2016 (21). In Canada, isolates resistant to ampicillin were more commonly found in 2011–2014 than in 2007–2010 with 52.5% of isolates showing these resistance markers (25). A previous survey on community-acquired respiratory bacteria in China reported that the resistance rate of *H. influenzae* isolates was higher in 2013–2014 than in 2009–2011 (26). Meanwhile, a multicenter surveillance of fastidious pathogens of the respiratory tract indicated that ampicillin-resistance rate was in accordance with β-lactamase production rate (31%) (27). A previous report in Chongqing, China, noted that *bla*_TEM−1_ encoding β-lactamase was the main mechanism of ampicillin-resistance of *H. influenzae* isolates. Moreover, diversity in the promoter, and the Prpt.b promoter may be related to high resistance of *H. influenzae* to ampicillin (14). The resistance rate of ampicillin in our study (2015–2018) was 45.10% which was higher than β-lactamase production rates of 33.3%. The emergence of more BLNAR isolates may contributed to the increase of ampicillin-resistance. Production of β-lactamase and mutation in PBP3 are two major mechanisms of resistance to ampicillin in *H. influenzae* (28). The diversity of TEM-1 gene in ampicillin-resistance might require a further research. Amino acid substitution in PBP3 is another important mechanism contributing to ampicillin-resistance, and different mutation sites in PBP3 might show different degrees of antimicrobial resistance. Three kinds of significant mutations near the conserved motif have been recognized: STVK (Ser-Thr-Val-Lys), SSN (Ser-Ser-Asn), and motif (Lys-Thr-Gly) (29). Isolates with significant PBP3 mutations were more resistant to β-lactam antibiotics (25). In this study, the rate of PBP3 mutation was up to 74.51% and isolates belonging to group III and II were prevalent in BLNAR and gBLNAR, respectively. MIC values of ampicillin, amoxillin-clavulanate and cefuroxime in group III were higher than other groups, which was also reported to contribute to the increase of the MICs of cefixime and cefuroxime by 10–60 times (9). In addition, BLNAR strains belonging to group I and II showed lower resistance to β-lactam antibiotics than those belonging to group III and III-like and high-BLNAR was predominant in Japan and Korea (30, 31). Three carbapenem-resistant *H. inluenzae* isolates were found in this study, which were rarely reported. Kazuki et al. found that amino acid in PBP3 of a isolates was altered with V525_N526insM insertion, and was responsible for the reduced susceptibility to carbapenems in *H. influenzae* (32). However, there was no special amino acid substitution of PBP3 in these three isolates in current study, which may suggest a third mechanism involved. AcrR is a repressor of the AcrAB efflux pump, which might contribute to β-lactam resistance. The mutation of acrR could also reduce carbapenem susceptibility (33).

Generally, the resistance of organisms to three or more different kinds of antimicrobials is defined as multidrug resistance (MDR) (34). No specific mechanism for multidrug resistance was found, it may be related to tradition mechanism, plasmid exchange and efflux pump. Only a small fraction of isolates (13.73%) in current study presented MDR with β-lactam-AZM-SXT as the most frequent pattern of resistance in this study. Although isolates resistant to fluoroquinolone were rare and only had been sporadically reported in Guangzhou, Hong Kong, and Taiwan (35), five isolates of current study were resistant to lomefloxacin but all susceptible to ciprofloxacin, which raised special concern for future epidemiological monitoring. Although β-lactamase and alteration in PBP3 reduced sensitivity of *H. influenzae* to β-lactam antibiotics, ciprofloxacin and third-generation cephalosporins resistance was not found indicating that they could be used in clinical treatment of *H. influenzae* infection among adult patients.

In conclusion, NTHi isolates with no dominant cloning became predominant in *H. influenzae* infections among adults in Shanghai, and cannot be prevented by Hib vaccine. Ampicillin and trimethoprim-sulfamethoxazole showed the lowest activity against *H. influenzae* isolates. PBP3 mutation and β-lactamase production were prevalent in *H. influenzae* strains and both contributed to ampicillin-resistance and reduced sensitivity to other β-lactam antibiotics in Shanghai, requiring a long-termed monitoring. A further multicenter surveillance of molecular epidemiology and antimicrobial susceptibility of *H. influenzae* may be required.

## Data Availability Statement

The datasets analyzed for this study can be found in the *Haemophilus influenzae* PubMLST database: https://pubmlst.org/bigsdb?db=pubmlst_hinfluenzae_isolates - the accession numbers are available within the Supplementary Material.

## Author Contributions

X-XL and S-ZX conceived and designed the experiments, performed the experiments, and wrote the paper. X-XL, S-ZX, F-FG, and W-PH analyzed the data. Y-XN and L-ZH contributed reagents, materials, and analysis tools.

### Conflict of Interest

The authors declare that the research was conducted in the absence of any commercial or financial relationships that could be construed as a potential conflict of interest. The reviewer YY declared a shared affiliation, with no collaboration, with several of the authors, X-XL, S-ZX, F-FG, W-PH, Y-XN, and L-ZH, to the handling editor at the time of review.
