# Supplementary material for: Molecular Epidemiology and Antimicrobial Resistance of Haemophilus influenzae in Adult Patients in Shanghai, China
Source: Front Public Health. 2020 Mar 27;8:95. doi: 10.3389/fpubh.2020.00095 (PMC7135888; doi:10.3389/fpubh.2020.00095)
Supplement: Supplementary file 1 [file Table_1.DOCX]

**Supplementary Table 1. Clinical information of adult patient with *H. influenzae* infection in Shanghai**

|  | number | percentage |
| --- | --- | --- |
| **Gender** |  |  |
| Female | 17 | 33.33% |
| Male | 34 | 66.67% |
| **Age (year)** |  |  |
| 19-64 | 30 | 58.82% |
| ≥65 | 21 | 41.18% |
| **Infection type^1^** |  |  |
| Invasive | 3 | 5.88% |
| Non-invasive | 48 | 94.12% |
| **Initial diagnosis** |  |  |
| Malignant tumor | 8 | 15.69% |
| Cardio-cerebrovascular disease | 7 | 13.73% |
| Pulmonary diseases | 5 | 9.80% |
| Autoimmune disease | 5 | 9.80% |
| Respiratory tract infections | 4 | 7.84% |
| Kidney disease | 3 | 5.88% |
| Hepatopathy | 3 | 5.88% |
| Other diseases^2^ | 8 | 15.69% |
| Undiagnosed | 8 | 15.69% |
| **Departments** |  |  |
| Pneumology | 12 | 23.53% |
| Dermatology | 7 | 13.73% |
| Nephropathy | 5 | 9.80% |
| Infections | 4 | 7.84% |
| Cardiothoracic surgery | 4 | 7.84% |
| Neurology | 3 | 5.88% |
| Intensive Care Unit (ICU) | 2 | 3.92% |
| Cardiology | 2 | 3.92% |
| Burn ward | 2 | 3.92% |
| Others^3^ | 10 | 19.61% |

1 A isolate separated from aseptic specimens was regarded as invasion, if not it was regarded as non-invasion.

2 Other diseases included hot crush injury, burn, eruptive pustulosis, fever, cushing's syndrome, anemia, eczema, acromegaly.

3 Others (wards) included radiation therapy ward, emergency,  traditional chinese medicine and so on.
